# 1,4-Bis(4-chloro­phen­yl)butane-1,4-dione

**DOI:** 10.1107/S1600536808039251

**Published:** 2008-11-29

**Authors:** Shuqin Li, Huisheng Li

**Affiliations:** a Department of Chemistry and Biology, Xiangfan University, Xiangfan 441053, People’s Republic of China

## Abstract

The mol­ecule of title compound, C_16_H_12_Cl_2_O_2_, is centrosymmetric. Thus, the asymmetric unit comprises two half-mol­ecules. The two benzene rings are coplanar in each independent molecule (dihedral angles = 0°). The crystal packing exhibits inter­molecular C—H⋯O hydrogen bonds and C—H⋯π inter­actions.

## Related literature

For applications of the title compound, see: Rao *et al.* (2004 ▶); Stauffer & Neier (2000 ▶); Shridhar *et al.* (1982 ▶). For the preparation of the title compound, see: Stetter (1976 ▶); Nimgirawath *et al.* (1976 ▶); Yamamoto *et al.* (2003 ▶); Yuguchi *et al.* (2004 ▶).
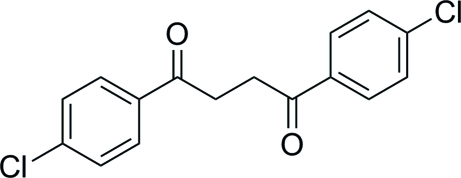


## Experimental

### 

#### Crystal data

C_16_H_12_Cl_2_O_2_
*M* *_r_* = 307.16
Monoclinic, 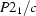

*a* = 10.3663 (2) Å
*b* = 5.2532 (1) Å
*c* = 26.1125 (6) Å
β = 95.272 (2)°
*V* = 1415.97 (5) Å^3^
*Z* = 4
Mo *K*α radiation
μ = 0.46 mm^−1^
*T* = 298 (2) K
0.20 × 0.10 × 0.10 mm

#### Data collection

Bruker SMART CCD area-detector diffractometer
Absorption correction: multi-scan (*SADABS*; Sheldrick, 1997 ▶) *T*_min_ = 0.914, *T*_max_ = 0.956
14170 measured reflections
2789 independent reflections
1836 reflections with *I* > 2σ(*I*)
*R*_int_ = 0.058

#### Refinement

*R*[*F* ^2^ > 2σ(*F* ^2^)] = 0.042
*wR*(*F* ^2^) = 0.117
*S* = 0.97
2789 reflections
181 parameters
H-atom parameters constrained
Δρ_max_ = 0.20 e Å^−3^
Δρ_min_ = −0.23 e Å^−3^

### 

Data collection: *SMART* (Bruker, 2001 ▶); cell refinement: *SAINT* (Bruker, 1999 ▶); data reduction: *SAINT*; program(s) used to solve structure: *SHELXS97* (Sheldrick, 2008 ▶); program(s) used to refine structure: *SHELXL97* (Sheldrick, 2008 ▶); molecular graphics: *SHELXTL* (Sheldrick, 2008 ▶); software used to prepare material for publication: *SHELXTL*.

## Supplementary Material

Crystal structure: contains datablocks I, global. DOI: 10.1107/S1600536808039251/kp2197sup1.cif

Structure factors: contains datablocks I. DOI: 10.1107/S1600536808039251/kp2197Isup2.hkl

Additional supplementary materials:  crystallographic information; 3D view; checkCIF report

## Figures and Tables

**Table 1:** Hydrogen-bond geometry (Å, °)

*D*—H⋯*A*	*D*—H	H⋯*A*	*D*⋯*A*	*D*—H⋯*A*
C8—H8*B*⋯O1^i^	0.97	2.59	3.553 (3)	173
C11—H11⋯O1^ii^	0.93	2.50	3.246 (2)	138
C8—H8*A*⋯*Cg*1^iii^	0.97	2.98	3.876 (2)	154

Symmetry codes: (i) 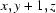; (ii) 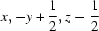; (iii) 
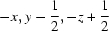
. *Cg*1 is the centroid of C9–C14.

**Fig. 1.**
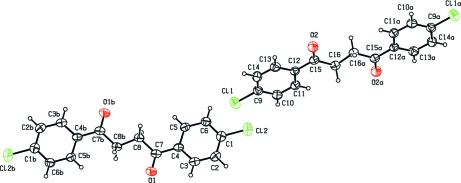
View of the title molecule showing the atom-labelling scheme. The displacement ellipsoids are drawn at the 30% probability level. H atoms are represented by spheres of arbitrary radius.

## supplementary crystallographic information

### Comment

1,4-Diketones are very useful intermediates for the synthesis of substituted furans, pyrroles and thiophenes *via* Paal-Knorr cyclization reaction (Rao *et al.*, 2004; Stauffer *et al.*, 2000; Shridhar *et al.*, 1982). A variety of methods have been reported for the preparation of these 1,4-dicarbonyl compounds (Stetter *et al.*, 1976; Yamamoto *et al.*, 2003; Yuguchi *et al.*, 2004).

The molecule is centrosymmetric. There are two halves of the molecules in the asymmetric unit (Fig. 1). The two phenyl rings are co-planar in both molecules. Intermolecular C—H···O hydrogen bonds and C—H···π interactions  stabilize the crystal packing (Table 1).

### Experimental

The title compound was synthesized as previously described by Nimgirawath *et al.* (1976). Colourless crystals suitable for X-ray data collection were obtained by slow evaporation of a 1:2 (*v*/*v*) ratio CH_2_Cl_2_:CH_3_OH solution at 293 K.

### Refinement

All H atoms were refine independently; C—H (methyl H) = 0.96 Å, C—H (methine H) = 0.93Å and 0.93 Å for thiophene H. The constraint *U*_iso_(H) = 1.2*U*_eq_(C) or 1.5*U*_eq_ (methyl C) was applied.

### Crystal data

**Table:**

C_16_H_12_Cl_2_O_2_	*F*_000_ = 632
*M**_r_* = 307.16	*D*_x_ = 1.441 Mg m^−^^3^
Monoclinic, *P*2_1_/*c*	Mo *K*α radiation λ = 0.71073 Å
Hall symbol: -P 2ybc	Cell parameters from 3417 reflections
*a* = 10.3663 (2) Å	θ = 2.6–23.1º
*b* = 5.25320 (10) Å	µ = 0.46 mm^−^^1^
*c* = 26.1125 (6) Å	*T* = 298 (2) K
β = 95.272 (2)º	Block, colourless
*V* = 1415.97 (5) Å^3^	0.20 × 0.10 × 0.10 mm
*Z* = 4	

### Data collection

**Table:**

Bruker SMART CCD area-detector diffractometer	2789 independent reflections
Radiation source: fine-focus sealed tube	1836 reflections with *I* > 2σ(*I*)
Monochromator: graphite	*R*_int_ = 0.058
*T* = 298(2) K	θ_max_ = 26.0º
φ and ω scans	θ_min_ = 1.6º
Absorption correction: multi-scan(SADABS; Sheldrick, 1997)	*h* = −12→12
*T*_min_ = 0.914, *T*_max_ = 0.956	*k* = −6→6
14170 measured reflections	*l* = −31→32

### Refinement

**Table:**

Refinement on *F*^2^	Secondary atom site location: difference Fourier map
Least-squares matrix: full	Hydrogen site location: inferred from neighbouring sites
*R*[*F*^2^ > 2σ(*F*^2^)] = 0.042	H-atom parameters constrained
*wR*(*F*^2^) = 0.117	*w* = 1/[σ^2^(*F*_o_^2^) + (0.0637*P*)^2^] where *P* = (*F*_o_^2^ + 2*F*_c_^2^)/3
*S* = 0.97	(Δ/σ)_max_ = 0.001
2789 reflections	Δρ_max_ = 0.21 e Å^−^^3^
181 parameters	Δρ_min_ = −0.23 e Å^−^^3^
Primary atom site location: structure-invariant direct methods	Extinction correction: none

### Special details

**Table:**

Geometry. All e.s.d.'s (except the e.s.d. in the dihedral angle between two l.s. planes) are estimated using the full covariance matrix. The cell e.s.d.'s are taken into account individually in the estimation of e.s.d.'s in distances, angles and torsion angles; correlations between e.s.d.'s in cell parameters are only used when they are defined by crystal symmetry. An approximate (isotropic) treatment of cell e.s.d.'s is used for estimating e.s.d.'s involving l.s. planes.
Refinement. Refinement of *F*^2^ against ALL reflections. The weighted *R*-factor *wR* and goodness of fit *S* are based on *F*^2^, conventional *R*-factors *R* are based on *F*, with *F* set to zero for negative *F*^2^. The threshold expression of *F*^2^ > σ(*F*^2^) is used only for calculating *R*-factors(gt) *etc*. and is not relevant to the choice of reflections for refinement. *R*-factors based on *F*^2^ are statistically about twice as large as those based on *F*, and *R*- factors based on ALL data will be even larger.

### Fractional atomic coordinates and isotropic or equivalent isotropic displacement parameters (Å^2^)

**Table:**

	*x*	*y*	*z*	*U*_iso_*/*U*_eq_	
C1	0.3102 (2)	0.4151 (4)	0.32315 (8)	0.0609 (5)	
C2	0.3437 (2)	0.2160 (4)	0.35628 (8)	0.0658 (6)	
H2	0.4069	0.0995	0.3488	0.079*	
C3	0.2824 (2)	0.1933 (4)	0.40024 (8)	0.0612 (6)	
H3	0.3041	0.0584	0.4225	0.073*	
C4	0.18851 (19)	0.3661 (3)	0.41264 (8)	0.0524 (5)	
C5	0.1579 (2)	0.5660 (4)	0.37840 (8)	0.0590 (5)	
H5	0.0960	0.6851	0.3859	0.071*	
C6	0.2175 (2)	0.5899 (4)	0.33398 (8)	0.0627 (6)	
H6	0.1955	0.7228	0.3113	0.075*	
C7	0.1272 (2)	0.3362 (4)	0.46133 (8)	0.0559 (5)	
C8	0.0298 (2)	0.5308 (4)	0.47540 (7)	0.0576 (5)	
H8A	−0.0386	0.5432	0.4476	0.069*	
H8B	0.0719	0.6955	0.4790	0.069*	
C9	0.1559 (2)	0.7318 (4)	0.15908 (8)	0.0593 (5)	
C10	0.1743 (2)	0.6200 (4)	0.11264 (8)	0.0623 (6)	
H10	0.1275	0.4755	0.1019	0.075*	
C11	0.2621 (2)	0.7227 (4)	0.08217 (8)	0.0575 (5)	
H11	0.2745	0.6459	0.0509	0.069*	
C12	0.33228 (18)	0.9383 (3)	0.09714 (7)	0.0508 (5)	
C13	0.3118 (2)	1.0476 (4)	0.14417 (8)	0.0628 (6)	
H13	0.3581	1.1926	0.1549	0.075*	
C14	0.2249 (2)	0.9467 (4)	0.17514 (8)	0.0674 (6)	
H14	0.2127	1.0220	0.2066	0.081*	
C15	0.42674 (19)	1.0574 (4)	0.06459 (7)	0.0537 (5)	
C16	0.45259 (19)	0.9285 (4)	0.01517 (8)	0.0558 (5)	
H16A	0.3713	0.9095	−0.0061	0.067*	
H16B	0.4865	0.7594	0.0229	0.067*	
Cl1	0.04740 (6)	0.59996 (12)	0.19828 (2)	0.0828 (2)	
Cl2	0.38806 (6)	0.44975 (15)	0.26764 (2)	0.0908 (3)	
O1	0.15594 (16)	0.1582 (3)	0.48988 (6)	0.0830 (5)	
O2	0.48192 (15)	1.2536 (3)	0.07810 (6)	0.0746 (5)	

### Atomic displacement parameters (Å^2^)

**Table:**

	*U*^11^	*U*^22^	*U*^33^	*U*^12^	*U*^13^	*U*^23^
C1	0.0561 (13)	0.0641 (12)	0.0619 (13)	−0.0073 (11)	0.0012 (10)	−0.0094 (10)
C2	0.0571 (13)	0.0598 (13)	0.0800 (15)	0.0045 (11)	0.0034 (12)	−0.0096 (12)
C3	0.0596 (14)	0.0472 (11)	0.0746 (14)	0.0029 (10)	−0.0055 (11)	0.0030 (10)
C4	0.0524 (12)	0.0432 (10)	0.0596 (12)	−0.0020 (9)	−0.0061 (10)	0.0006 (9)
C5	0.0639 (14)	0.0499 (11)	0.0632 (13)	0.0086 (10)	0.0063 (10)	0.0010 (10)
C6	0.0705 (15)	0.0559 (12)	0.0609 (13)	0.0026 (11)	0.0023 (11)	0.0044 (10)
C7	0.0566 (13)	0.0454 (11)	0.0637 (13)	−0.0034 (9)	−0.0049 (10)	0.0052 (9)
C8	0.0648 (14)	0.0473 (11)	0.0600 (12)	−0.0027 (10)	0.0017 (10)	0.0039 (9)
C9	0.0569 (13)	0.0607 (12)	0.0600 (13)	0.0043 (10)	0.0036 (10)	0.0077 (10)
C10	0.0634 (14)	0.0543 (12)	0.0682 (14)	−0.0080 (10)	0.0007 (11)	−0.0027 (10)
C11	0.0614 (13)	0.0531 (11)	0.0575 (12)	−0.0005 (10)	0.0036 (10)	−0.0074 (9)
C12	0.0530 (12)	0.0478 (10)	0.0504 (11)	0.0042 (9)	−0.0017 (9)	−0.0011 (9)
C13	0.0733 (15)	0.0528 (12)	0.0609 (13)	−0.0079 (11)	−0.0011 (11)	−0.0067 (10)
C14	0.0807 (16)	0.0673 (14)	0.0543 (12)	−0.0008 (12)	0.0064 (11)	−0.0065 (11)
C15	0.0502 (12)	0.0520 (11)	0.0577 (12)	0.0030 (10)	−0.0020 (9)	−0.0021 (9)
C16	0.0504 (12)	0.0559 (11)	0.0599 (12)	0.0011 (9)	−0.0012 (9)	−0.0014 (9)
Cl1	0.0788 (5)	0.0941 (5)	0.0772 (4)	−0.0099 (3)	0.0169 (3)	0.0112 (3)
Cl2	0.0818 (5)	0.1167 (6)	0.0764 (4)	0.0026 (4)	0.0215 (3)	−0.0058 (4)
O1	0.0957 (13)	0.0697 (10)	0.0855 (11)	0.0224 (9)	0.0175 (9)	0.0298 (9)
O2	0.0803 (11)	0.0631 (9)	0.0819 (11)	−0.0212 (8)	0.0150 (8)	−0.0150 (8)

### Geometric parameters (Å, °)

**Table:**

C1—C6	1.377 (3)	C9—C14	1.381 (3)
C1—C2	1.381 (3)	C9—Cl1	1.733 (2)
C1—Cl2	1.732 (2)	C10—C11	1.373 (3)
C2—C3	1.368 (3)	C10—H10	0.9300
C2—H2	0.9300	C11—C12	1.383 (3)
C3—C4	1.391 (3)	C11—H11	0.9300
C3—H3	0.9300	C12—C13	1.390 (3)
C4—C5	1.397 (3)	C12—C15	1.492 (3)
C4—C7	1.481 (3)	C13—C14	1.371 (3)
C5—C6	1.369 (3)	C13—H13	0.9300
C5—H5	0.9300	C14—H14	0.9300
C6—H6	0.9300	C15—O2	1.215 (2)
C7—O1	1.216 (2)	C15—C16	1.503 (3)
C7—C8	1.506 (3)	C16—C16^ii^	1.517 (4)
C8—C8^i^	1.511 (4)	C16—H16A	0.9700
C8—H8A	0.9700	C16—H16B	0.9700
C8—H8B	0.9700	Cl1—Cl2	3.8985 (9)
C9—C10	1.376 (3)		
			
C6—C1—C2	121.1 (2)	C10—C9—Cl1	120.01 (17)
C6—C1—Cl2	119.31 (17)	C14—C9—Cl1	119.58 (16)
C2—C1—Cl2	119.60 (18)	C11—C10—C9	119.75 (19)
C3—C2—C1	118.8 (2)	C11—C10—H10	120.1
C3—C2—H2	120.6	C9—C10—H10	120.1
C1—C2—H2	120.6	C10—C11—C12	121.17 (19)
C2—C3—C4	121.84 (19)	C10—C11—H11	119.4
C2—C3—H3	119.1	C12—C11—H11	119.4
C4—C3—H3	119.1	C11—C12—C13	117.95 (19)
C3—C4—C5	117.68 (19)	C11—C12—C15	122.57 (17)
C3—C4—C7	119.70 (18)	C13—C12—C15	119.47 (17)
C5—C4—C7	122.62 (19)	C14—C13—C12	121.59 (19)
C6—C5—C4	121.17 (19)	C14—C13—H13	119.2
C6—C5—H5	119.4	C12—C13—H13	119.2
C4—C5—H5	119.4	C13—C14—C9	119.2 (2)
C5—C6—C1	119.39 (19)	C13—C14—H14	120.4
C5—C6—H6	120.3	C9—C14—H14	120.4
C1—C6—H6	120.3	O2—C15—C12	120.42 (18)
O1—C7—C4	120.50 (19)	O2—C15—C16	120.99 (19)
O1—C7—C8	120.30 (19)	C12—C15—C16	118.58 (17)
C4—C7—C8	119.20 (16)	C15—C16—C16^ii^	113.6 (2)
C7—C8—C8^i^	113.5 (2)	C15—C16—H16A	108.8
C7—C8—H8A	108.9	C16^ii^—C16—H16A	108.8
C8^i^—C8—H8A	108.9	C15—C16—H16B	108.8
C7—C8—H8B	108.9	C16^ii^—C16—H16B	108.8
C8^i^—C8—H8B	108.9	H16A—C16—H16B	107.7
H8A—C8—H8B	107.7	C9—Cl1—Cl2	75.29 (7)
C10—C9—C14	120.4 (2)	C1—Cl2—Cl1	86.46 (7)
			
C6—C1—C2—C3	0.5 (3)	C10—C11—C12—C13	0.3 (3)
Cl2—C1—C2—C3	179.23 (15)	C10—C11—C12—C15	−178.93 (18)
C1—C2—C3—C4	−0.6 (3)	C11—C12—C13—C14	0.0 (3)
C2—C3—C4—C5	0.2 (3)	C15—C12—C13—C14	179.26 (19)
C2—C3—C4—C7	−178.57 (18)	C12—C13—C14—C9	−0.2 (3)
C3—C4—C5—C6	0.5 (3)	C10—C9—C14—C13	0.2 (3)
C7—C4—C5—C6	179.23 (18)	Cl1—C9—C14—C13	179.13 (16)
C4—C5—C6—C1	−0.7 (3)	C11—C12—C15—O2	176.53 (19)
C2—C1—C6—C5	0.2 (3)	C13—C12—C15—O2	−2.7 (3)
Cl2—C1—C6—C5	−178.55 (15)	C11—C12—C15—C16	−3.9 (3)
C3—C4—C7—O1	−1.9 (3)	C13—C12—C15—C16	176.85 (17)
C5—C4—C7—O1	179.45 (18)	O2—C15—C16—C16^ii^	−2.1 (3)
C3—C4—C7—C8	177.44 (18)	C12—C15—C16—C16^ii^	178.39 (19)
C5—C4—C7—C8	−1.2 (3)	C10—C9—Cl1—Cl2	107.86 (17)
O1—C7—C8—C8^i^	−3.6 (3)	C14—C9—Cl1—Cl2	−71.11 (16)
C4—C7—C8—C8^i^	177.0 (2)	C6—C1—Cl2—Cl1	−43.73 (16)
C14—C9—C10—C11	0.1 (3)	C2—C1—Cl2—Cl1	137.48 (16)
Cl1—C9—C10—C11	−178.83 (15)	C9—Cl1—Cl2—C1	158.48 (10)
C9—C10—C11—C12	−0.4 (3)		

Symmetry codes: (i) −*x*, −*y*+1, −*z*+1; (ii) −*x*+1, −*y*+2, −*z*.

### Hydrogen-bond geometry (Å, °)

**Table:**

*D*—H···*A*	*D*—H	H···*A*	*D*···*A*	*D*—H···*A*
C8—H8B···O1^iii^	0.97	2.59	3.553 (3)	173
C11—H11···O1^iv^	0.93	2.50	3.246 (2)	138
C8—H8A···Cg1^v^	0.97	2.98	3.876 (2)	154

Symmetry codes: (iii) *x*, *y*+1, *z*; (iv) *x*, −*y*+1/2, *z*−1/2; (v) −*x*, *y*−1/2, −*z*+1/2.
